# A rare case of spontaneous aortic thrombosis presenting with unilateral auto-Lisfranc joint amputation in a young adult: A case report

**DOI:** 10.1016/j.ijscr.2025.111472

**Published:** 2025-06-03

**Authors:** Ayto Addisu Negash, Amanuel Dagabas Wakoya, Dagmawi Anteneh Teferi, Hanna Tsehay Abebe, Rakeb Mulugeta Feyissa, Amanuel Mesfin Oljira

**Affiliations:** a Department of Emergency Medicine and Critical Care, St. Paul's Hospital Millennium Medical College, Addis Ababa, Ethiopia; b Department of Surgery, St. Paul's Hospital Millennium Medical College, Addis Ababa, Ethiopia; c Department of Internal Medicine, St. Paul's Hospital Millennium Medical College, Addis Ababa, Ethiopia; d Department of General Surgery, Ambo University Referral Hospital, Oromia, Ethiopia

**Keywords:** Aortic thrombosis, Critical limb ischemia, Lisfranc joint amputation, Young adult, Vascular disease, Case report

## Abstract

**Introduction and importance:**

Spontaneous aortic thrombosis is a rare clinical entity in young adults. Its rapid progression from intermittent claudication to critical limb ischemia presents significant diagnostic and therapeutic challenges. Early recognition and timely intervention are crucial to prevent severe complications. We report a rare case of spontaneous aortic thrombosis that presented with unilateral auto-Lisfranc joint amputation in a young adult.

**Case presentation:**

A 22-year-old male presented with progressive intermittent claudication lasting five months, and a week's history of dark skin discoloration in both feet, leading to a left auto-Lisfranc joint amputation. Imaging studies revealed severe abdominal aortic thrombosis with absent blood flow to the lower extremities. Following this, an aorto-bifemoral bypass procedure was performed, resulting in an improved condition during discharge.

**Clinical discussion:**

This case underscores the atypical presentation of primary aortic thrombosis in a young adult with no identifiable risk factors. The striking occurrence of unilateral auto-Lisfranc joint amputation as the initial symptom is exceptionally rare. Imaging revealed extensive aortic thrombus without indications of vasculitis or an embolic source, thereby necessitating surgical revascularization.

**Conclusion:**

Early diagnosis and prompt intervention are crucial in preventing severe morbidity and limb loss related to acute limb ischemia. This case exemplifies the necessity for healthcare professionals to maintain heightened awareness of vascular diseases, particularly in young adults, and to act decisively to ensure optimal patient outcomes. Continuous follow-up, education on identifying symptoms of thromboembolism, and strict adherence to anticoagulation therapy are essential components in improving long-term prognosis and preventing recurrence.

## Highlights

• A high index of suspicion is needed to diagnose arterial thrombosis in young adult.
• The rapidly progressing acute limb ischemia resulting in Auto Lisfranc amputation underscores the dangers of delayed diagnosis.
• Early detection and surgical intervention of arterial thrombosis can prevent limb loss.
• Thrombectomy and vascular bypass grafts are the options for the management of arterial thrombosis to salvage a limb.

## 1 Introduction

### 1.1 Background

Arterial embolism is a common clinical entity, with over 80 % of emboli originating from the heart due to structural or functional abnormalities such as atrial fibrillation, valvular disease, or myocardial infarction. In contrast, thrombosis involves the in situ formation of blood clots within the arterial system and is typically associated with underlying vascular disease. Aortic thrombosis is a less common cause of arterial occlusion and generally occurs in the presence of significant aortic pathology, including atherosclerosis, aneurysms, dissections, trauma, infections, or tumors [1]. Rarely, a thrombus may form in an otherwise macroscopically normal aorta, a condition termed primary aortic thrombosis. The pathogenesis of this condition remains unclear but has been linked to hypercoagulable states, malignancy, iatrogenic pharmacotherapy, inflammatory disorders, and recreational drug use [2].

The clinical consequences of aortic thrombosis are predominantly embolic. Depending on the destination of the emboli, organ-specific symptoms can arise—for instance, renal embolization may lead to acute kidney injury [3], splenic emboli may result in left upper quadrant pain or complications such as infarction, abscess, or hemorrhage [4], and lower limb embolization may cause acute limb ischemia (ALI). ALI is associated with amputation and mortality rates of 13–15 % and 9–12 %, respectively [5], and may progress to chronic limb-threatening ischemia (CLTI), characterized by ischemic rest pain, ulceration, and gangrene.

### 1.2 Rationale

We report a rare and atypical case of spontaneous auto-amputation at the Lisfranc joint, secondary to CLTI resulting from primary aortic thrombosis in a young adult without predisposing cardiovascular or systemic disease. Although ALI typically presents unilaterally, bilateral lower limb ischemia has been reported in rare instances in younger patients [6]. Moreover, symptoms may be vague, gradual, or initially misinterpreted as musculoskeletal pain, further complicating diagnosis [7]. In our case, this led to a delayed diagnosis, and the ischemic process progressed to auto-amputation of the forefoot—a previously undocumented outcome of aortic thrombosis. This case is significant for its rarity, atypical presentation, and serious clinical outcome, underlining the importance of early vascular evaluation and a high index of suspicion for thrombotic disorders in otherwise healthy young individuals.

Guidelines and Literature: Diagnostic workup for arterial occlusion typically involves bedside Doppler ultrasonography as an initial tool, with computed tomography angiography (CTA) considered the gold standard for identifying aortic thrombi and associated embolic phenomena [8]. Aortic thrombi are generally associated with underlying structural disease [9], and thrombosis in a grossly normal aorta, particularly in a young patient, warrants extensive evaluation for thrombophilia. Common prothrombotic conditions include Factor V Leiden mutation, which is the most frequent heritable thrombophilia, as well as elevated homocysteine levels and deficiencies in protein C and protein S [10]. Management usually involves initial anticoagulation therapy to prevent thrombus propagation and embolization. In persistent or recurrent embolism cases, thrombolysis or surgical intervention may be required, with treatment tailored to the patient's risk profile and clinical presentation [11].

To the best of our knowledge, this is the first documented case of Lisfranc-level auto-amputation as a consequence of primary aortic thrombosis. This report aims to expand awareness of such atypical vascular presentations and promote early diagnostic vigilance in similar cases.

This case report has been reported in line with the Updated and Revised 2025 SCARE guidelines [12].

## 2 Case presentation

**Fig. 1:**
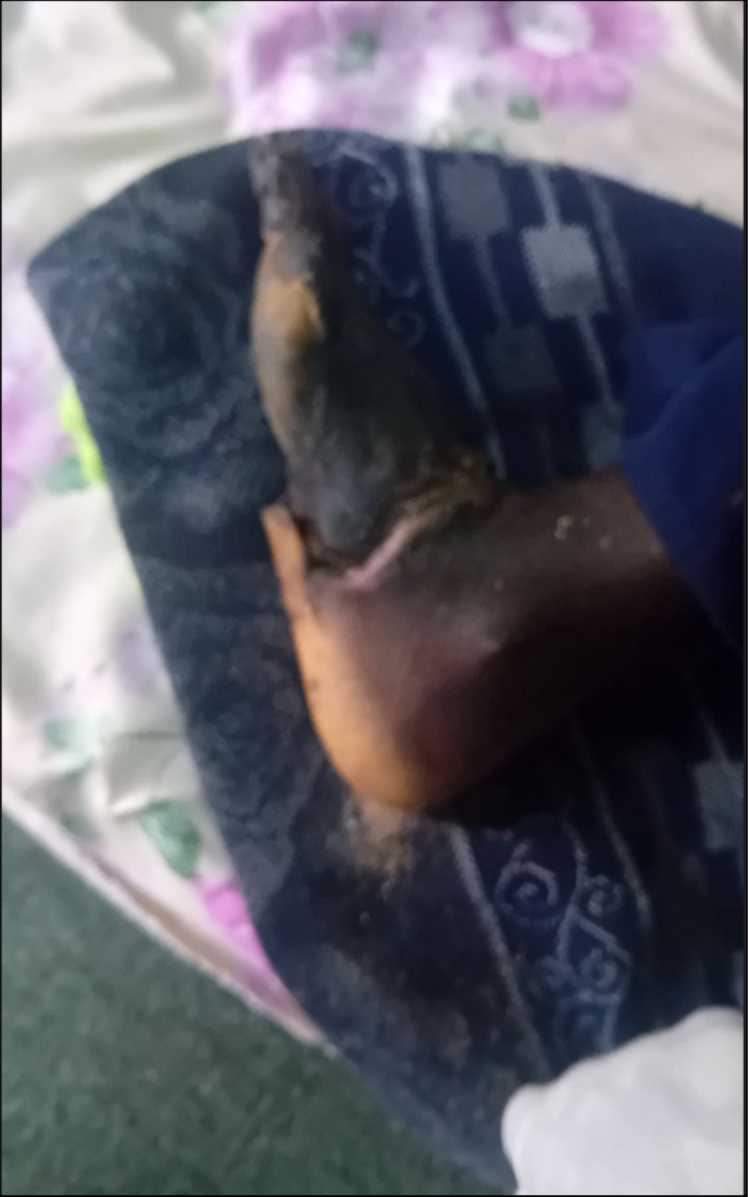
The left foot exhibiting necrotic, darkened tissue below mid-foot, accompanied by sloughing of the surrounding tissue.

A 22-year-old male presented with a complaint of left foot tissue loss distal to midfoot, which had occurred two days before his visit. Upon further inquiry, he reported experiencing intermittent claudication in his left leg and foot for the past five months. This discomfort progressively worsened over time, becoming more intense as the months passed. About three weeks ago, he noticed skin hyperpigmentation on his left foot, with a more pronounced discoloration on the left foot. One week before his presentation, he took a photo of his left foot, which showed gangrenous tissue distal to the mid-foot, along with sloughing of the surrounding tissue (Fig. 1). Additionally, the patient noted that his right foot had also started to hyperpigment over the past week, accompanied by pain. In response to these symptoms, the patient sought treatment from traditional healers and used holy water for a week; however, there was no improvement in his condition. Otherwise, the patient had no history of trauma, surgery, smoking, drug use, or malignancy, The patient has no history of peripheral artery disease (PAD) or coronary artery disease (CAD), diabetes, hypertension, or hyperlipidemia. He also had no previous consultations, referrals, or treatments for similar complaints before the current presentation. Additionally, there was no family history of similar vascular issues.

**Fig. 2:**
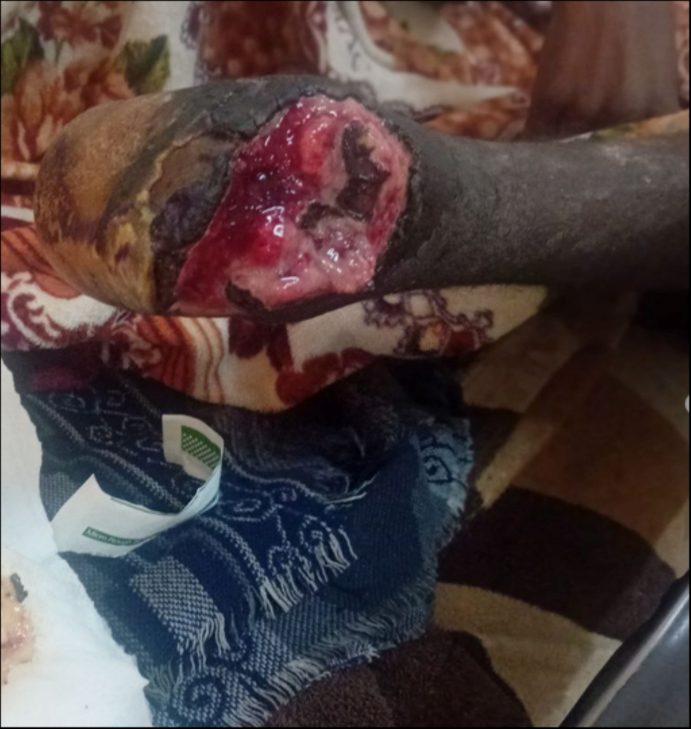
The image depicts a wound exhibiting central necrosis, and surrounding necrotic tissue with complete loss of tissue below left mid-foot.

A physical examination revealed normal vital signs; however, the patient appeared emaciated. Significant findings included an amputated left midfoot, with the wound exhibiting central necrosis and surrounding necrotic tissue, along with a complete loss of tissue beneath the joint (Fig. 2). The pulses in the right dorsalis pedis, right posterior tibialis, popliteal artery, and right femoral artery were palpable but weak. In contrast, left posterior tibialis and left popliteal artery pulses were not palpable, while the left femoral pulse was palpable, but weak.

Based on the clinical history and examination findings, critical limb ischemia with superinfection was entertained. The patient was started on unfractionated heparin (UFH), receiving a loading dose of 5000 IU intravenously (IV), followed by 12,500 IU subcutaneously (SC) twice daily, ceftriaxone 1 g (gm) IV twice daily, and metronidazole 500 mg (mg) IV three times per day. Additionally, 50 mg of tramadol was administered IV for pain management, and wound care was done.

Laboratory investigations showed elevated C-reactive protein (CRP) levels at 185.1 mg/L. Upon complete blood count, White blood cells were within normal range and no shifting, moderate anemia, indicated by a hemoglobin level of 8.4 g/dl and normal platelet count. Renal function tests (RFT), liver function tests (LFT), and electrolyte levels were all within the normal range. Further tests like thrombophilia, autoimmune, vasculitis workups, and tumor markers were not available on-site, and referral to an external lab was not completed due to financial constraints. However, imaging revealed no signs of malignancy (see Table 1).

**Table 1:** Laboratory results of the patient upon initial presentation.

Complete blood count	Result	Reference range
White blood cell	8,6	3.9–10.1 × 10^3^/mm^3^
Neutrophil	76.0	30.4–74.6 %
Lymphocyte	13.9	17.8–61.5 %
Hemoglobin	8.4	10.4–14.7 g/dl
Hematocrit	26.7	34.4–48.3 %
Mean cell volume	77.6	74.3–98.3 fl
Mean cell hemoglobin	31.5	25.7–33.6 pg.
Platelet count	410	100–300 × 10^3^/μL
Erythrocyte sedimentation rate	10	0–20 mm/h
PICT	Non-reactive	
C-reactive protein	185.1	<10 mg/L
Qualitative ANA	Non-reactive	
Random blood sugar	109	70–140 mg/dl
Creatinine	0.7	0.7–1.3 mg/dl
Urea	28.7	16.6–48.5 mg/dl
Sodium	137	136–145 mmol/l
Potassium	4.3	3.5–5.5 mmol/l
Calcium (ionized)	1.1	1.05–1.35 mmol/l
Magnesium	2.2	1.9–2.5 mg/dl
Coagulation profile		
INR	1.1	
PT	18 s	9.9–25 s
aPTT	22 s	24–40 s
Liver function test		
AST	9.4 IU/l	0–40 IU/L
ALT	7.5 IU/l	0–45 IU/L
ALP	82 Mg/dl	0–275 Mg/dl

**Fig. 3:**
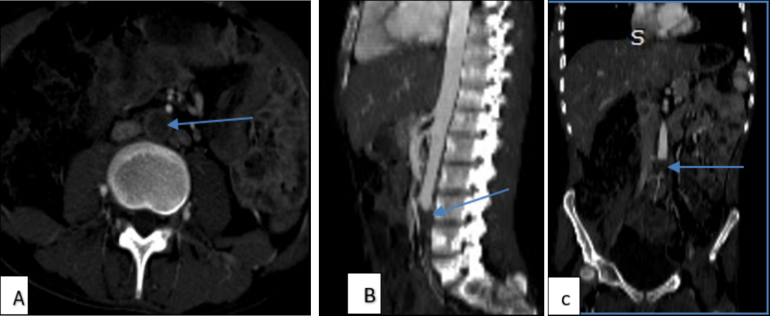
A) Axial, B) sagittal, and C) coronal views of abdominal CT angiography show a hypodense filling defect located in the distal abdominal aorta infra renal at the L3 level and absence of flow below the defect.

**Fig. 4:**
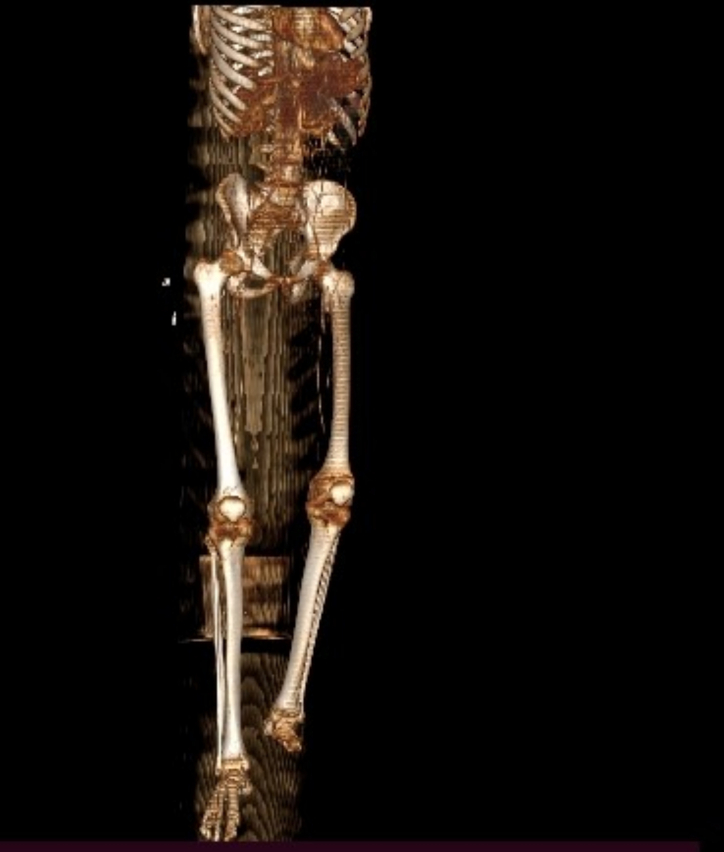
Three-dimensional radiograph image showing left mid-foot disarticulation.

Electrocardiography shows a normal sinus rhythm and normal echocardiography as well. Doppler ultrasound revealed bilateral occlusions of the common iliac artery (CIA) and the common femoral artery (CFA), with absent blood flow in most lower extremity arteries. The only noted flow was monophasic in the right posterior tibial artery. Furthermore, an abdominal CTA confirmed thrombosis of the distal infrarenal abdominal aorta, with the absence of flow in its first branches bilaterally (Fig. 3A, B, C). The three-dimensional (3D) view of lower extremity CTA also shows the left mid-foot disarticulation (Fig. 4).

After completing the investigation and imaging, we confirmed an auto-Lisfranc joint amputation due to spontaneous aortic thrombosis. Consequently, the vascular surgery team was involved in the procedure. They performed an aortic-bifemoral bypass under general anesthesia. The intraoperative findings were consistent with the preoperative imaging results.

PICT Provider-Initiated Counseling and Testing ANA, Anti-Nuclear Antibody INR, international normalizing ratio PT, prothrombin aPTT, activated Partial Thromboplastin Time, AST Aspartate Aminotransferase ALT Alanine Aminotransferase, ALP Alkaline Phosphatase.

**Fig. 5:**
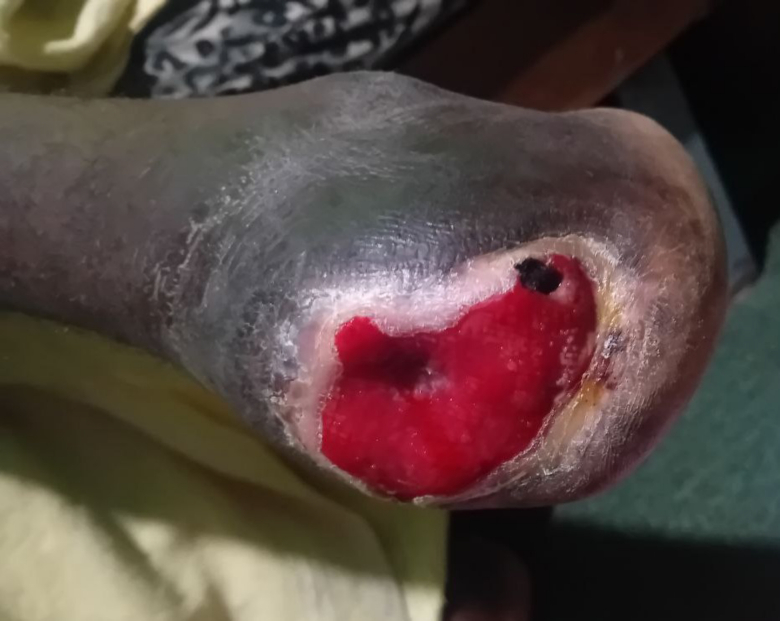
The wound has well-granulated tissue after one month of treatment.

**Fig. 6:**
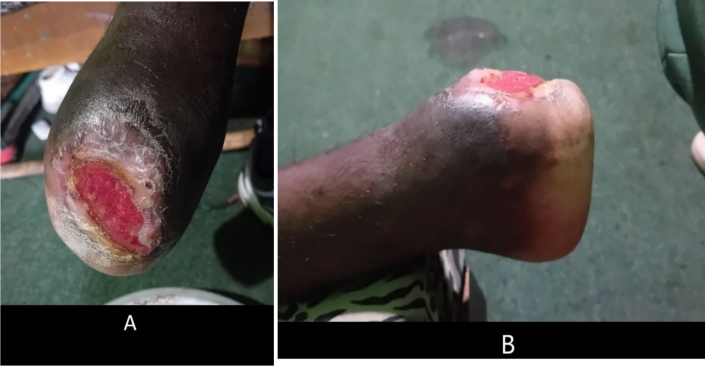
Granulated wound after 2 months of surgical intervention.

Following successful surgical intervention, the patient was discharged with rivaroxaban 15 mg orally twice daily for the first 3 weeks, and 10 mg orally once daily till now, plus aspirin 81 mg orally once daily for the first 1 month postoperatively and scheduled for follow-up one month later. During his follow-up appointment, he demonstrated significant improvement, with strong and palpable pulses in the right femoral, right popliteal, right posterior tibialis, and dorsalis pedis arteries. The left posterior tibial, left popliteal artery, and left femoral arteries had weak but still palpable pulses, and the wound also looked well-granulated (Fig. 5). At his second follow-up visit, two months post-surgery and one month after the initial follow-up, arterial pulses in both lower extremities were stronger. Wound healing improved significantly following the intervention and the initiation of pharmacotherapy. Additionally, his leg appeared pink compared to his previous skin discoloration (Fig. 6A, B).

## 3 Discussion

Primary thrombosis of the aorta was first described in the late 1940s [13] and was defined as a thrombus attached to the aortic wall in the absence of any atherosclerotic or aneurysmal disease in the aorta [2]. Thrombosis in a normal aorta (non-atherosclerotic and non-aneurysmal), although it is a rare condition, should still be taken into account, as demonstrated in our patient's case [2,14].

This case report highlights the rare occurrence of primary aortoiliac thrombosis in a young adult, presenting with critical limb ischemia and subsequent Lisfranc joint amputation. The patient's history of intermittent claudication, progressive foot discoloration, and eventual loss of the left foot underscores the importance of recognizing atypical vascular presentations in younger populations. Primary aortoiliac thrombosis is infrequently reported in individuals without significant risk factors, such as atherosclerosis or hypercoagulable states.

The clinical presentation aligns with existing literature that documents lower extremity complications due to aortoiliac occlusion. Studies indicate that young adults can experience sudden ischemic events despite the absence of traditional risk factors, emphasizing the need for vigilance in diagnosis and treatment [15]. In this case, limb ischemia manifested after months of progressive claudication in both legs, highlighting the importance of evaluating unusual presentations of arterial occlusion as noted in emerging literature. The progression from claudication to CLTI demonstrates how quickly vascular compromise can lead to severe outcomes, reinforcing the necessity for timely intervention.

Diagnostic imaging plays a crucial role in confirming the diagnosis of primary aortoiliac thrombosis. Doppler ultrasound and CTA are crucial for assessing vascular occlusions, as they provide vital information regarding blood flow and thrombus formation [9,16]. In our case, imaging revealed significant thrombus occlusion in the infrarenal abdominal aorta and its branches, but no findings suggesting large vessel vasculitis, like aortic wall thickening, which necessitated urgent surgical intervention. Additionally, the electrocardiogram showed a normal sinus rhythm.

It is also critical to consider any underlying conditions that may predispose younger patients to thrombotic events. Conditions such as Tumor markers, intraoperative thrombus sample for pathological analysis, antiphospholipid or inherited thrombophilia, and vasculitis should be investigated to prevent recurrence [10]. But in this case report, the above evaluations were not conducted due to their on-site unavailability and financial issues for the outside laboratory.

Although primary aortic thrombosis implies macroscopically normal aorta it is believed that underlying subclinical processes may underlie the thrombus formation. Therefore, it is essential for patients diagnosed with this condition to be referred to specialized facilities capable of conducting comprehensive evaluations like thrombophilia, autoimmune diseases [2]. The qualitative antinuclear antibody (ANA) test was non-reactive, and infectious causes of vasculitis, such as HIV and syphilis, were negative. Additionally, the initial abdominal CT scan and abdominal ultrasound did not show any intra-abdominal masses. The high level of inflammatory markers (elevated CRP) could represent the systemic inflammatory response to the gangrene.

There is no robust evidence to offer guidance for the treatment of aortic mural thrombus. Treatment modalities used with variable success for primary aortic thrombosis management include anticoagulation therapy alone, thrombolysis, thromboaspiration, and surgery [2,14]. When considering therapeutic options for thoracic aorta thrombi, it is necessary to view them as a heterogeneous group rather than a single entity, each having a different clinical course and prognosis depending on its nature and etiology [2].

While anticoagulation has been the main treatment approach and isolated case reports have demonstrated thrombus resolution with anticoagulation alone, studies indicate that the persistence of thrombus burden or the risk of recurrent embolism remains elevated (over 25 %) in comparison to open surgical thrombus removal, which has a lower risk of 9 % [9,17,18]. Recurrent embolism notably raises the likelihood of major amputation (9 % for those treated with anticoagulation alone compared to 2.3 % in the surgical group) and can lead to life-threatening visceral ischemia [17]. Additionally, a significant issue with relying solely on anticoagulation is that there is no established duration for treatment or optimal target range for the international normalized ratio [14].

Minimally invasive options like catheter aspiration and systemic or catheter-directed thrombolysis, although described with varying success rates, carry a high risk of distal embolization during the procedure itself and do not promise complete removal or exclusion of thrombus [19,20]. The preferred surgical approach is often thromboembolectomy, followed by thrombolysis or aorto-bifemoral bypass, as demonstrated in our patient; however, the choice depends on the etiology of the occlusion [21]. While embolectomy is more common for patients with an underlying embolic occlusion, bypass surgery is typically favored for those with in situ thrombosis [21]. Additionally, our patient continued to receive aspirin and unfractionated heparin postoperatively.

In our review, we identified two closely related case reports from the USA and Portugal involving female patients aged 46 and 49, respectively, both with a history of smoking. In these cases, the diagnosis was confirmed through CT angiography, and subsequent thrombophilia and autoimmune testing yielded no significant findings [2,4]. However, unlike our case, which presented solely with critical limb ischemia, both patients exhibited signs of intra-abdominal organ embolization, likely due to the location of the thrombus in the proximal descending aorta. Both cases were managed medically; the first was treated with warfarin, while the second received apixaban and aspirin. In contrast, our patient underwent thrombectomy in addition to medical therapy.

This case also highlights the importance of recognizing atypical presentations of arterial occlusion. The patient's reliance on traditional healing practices delayed appropriate medical intervention, emphasizing the need for increased awareness among healthcare providers regarding potential vascular emergencies and promoting health-seeking behavior among youth. Early recognition and prompt surgical intervention are critical in preventing significant morbidity and limb loss associated with CLTI.

The case report is limited by the lack of further workup to screen for underlying risk factors such as thrombophilia, tumor markers, and thrombus analysis through biopsy. These additional investigations could have provided more insight into the etiology of the spontaneous aortic thrombosis in young adults.

## CRediT authorship contribution statement

Ayto Addisu Negash: Study conceptualization and design, original draft write-up, data curation, paper review & editing, and patient management
Amanuel Dagabas Wakoya: Study conceptualization and design, original draft write-up, data curation, paper review & editing, and patient management
Dagmawi Anteneh Teferi: Study conceptualization and design, original draft write-up, data curation, paper review & editing, and patient management
Hanna Tsehay Abebe: Study conceptualization and design, original draft write-up, data curation, paper review & editing, and patient management
Rakeb Mulugeta Feyissa: study conceptualization and design, original draft write-up, data curation, paper review & editing, and patient management
Amanuel Mesfin Oljira: study conceptualization and design, original draft write-up, data curation, paper review & editing, and patient management.

## Consent

Written informed consent was obtained from the patient for publication of this case report and accompanying images. A copy of the written consent is available for review by the Editor-in-Chief of this journal on request.

## Ethical approval

Ethical approval is deemed unnecessary by St. Paul's Hospital Millennium Medical College Institutional Review Board as this is a single rare case faced during clinical practice and it doesn't involve experiments in humans or animals.

## Guarantor

Ayto Addisu Negash.

## Funding

This research did not receive any specific grant from funding agencies in the public, commercial, or not-for-profit sectors.

## Declaration of competing interest

The authors declare that they have no conflict of interest.
